# Oral health status and its impact on the quality of life of children and adolescents living with HIV-1

**DOI:** 10.1186/1756-0500-7-478

**Published:** 2014-07-28

**Authors:** Natália Spillere Rovaris, Dayani Galato, Fabiana Schuelter-Trevisol, Jane da Silva, Leandro da Silva Linhar, Daniela Alba Nickel, Jefferson Traebert

**Affiliations:** 1 Graduate Program in Health Sciences, University of Southern Santa Catarina, 88704-900 Tubarão, SC, Brazil; 2 Undergraduation in Pharmacy, University of Brasilia, 72220-900 Brasília, DF, Brazil

**Keywords:** Oral health, Oral health-related quality of life, Caries, Children, HIV

## Abstract

**Background:**

Oral health problems can generate considerable negative effects on the quality of life of individuals living with HIV. The aim of this study was investigate the oral health status and its impact on the quality of life of 1 to 18 years-old living with HIV-1 under follow-up at referral centers in Southern Brazil.

**Methods:**

A cross-sectional study involving individuals under follow-up (n = 36) was carried out. The individuals living with HIV-1 and their guardians underwent individual interviews using validated questionnaires for assessing oral health-related quality of life according to age group. Clinical oral examinations were performed to establish oral health status, in terms of caries and treatment need, HIV-1-related gingival as well as stomatological changes. Medical records were reviewed searching for clinical history of the infection and the presence of HIV-1-related diseases. Association studies between frequent/very frequent oral health-related impact on quality of life and independent variables were performed using Fisher’s exact test.

**Results:**

The prevalence of frequent/very frequent oral health-related impact on quality of life was 69.0%. The prevalence of caries was 75.9%. Gingival changes were present in 20.7% of the individuals. Dental treatment was needed in 72.4% of the patients. HIV-1-related disease was present in 55.2%. The variables significantly associated with the prevalence of frequent/very frequent impact on oral health-related quality of life were dental treatment need (p = 0.037) and being more than 12 years of age (p = 0.041).

**Conclusions:**

Individuals living with HIV-1 with need for dental treatment and those over 12-years of age reported a statistically higher frequency of frequent/very frequent oral health-related impact on quality of life.

## Background

Acquired Immunodeficiency Syndrome (AIDS) inflicts suffering to both patients and their careers, which extends beyond symptoms, limitations and disease-related treatment. This becomes more relevant in children and adolescents [1]. People living with Human Immunodeficiency Virus type 1 (HIV-1) may develop oral and systemic manifestations as a result of the natural history of the disease, which can cause a negative impact of quality of life [2]. In fact, oral lesions are among frequent opportunistic infections that occur in individuals living with HIV-1, with the majority developing some form of stomatological manifestation throughout the course of the infection [3,4].

Children living with HIV-1 may have an increased prevalence of dental caries and gingival problems as a result of sugary medications, antiretrovirals, a sugar-rich diet, inadequate hygiene habits and reduced salivary flow [5,6]. However, it is important to highlight that the use of antiretroviral therapy has contributed to significantly reduce oral manifestations of HIV-1 in the soft tissues [7]. Additionally, children present a primary viremia earlier in life because their immune system is relatively immature [8].

Brazil, via the National Health System (*SUS*) provides universal and free access to the medications used in the treatment of AIDS. Brazilian policies have well-established guidelines based on scientific evidence, with actions that reduce the risk of vertical transmission of HIV-1 [9]. Despite a well-established policy, little is known about the oral health status and its reflection on the quality of life of children and adolescents living with HIV-1 in Brazil. Buczinski et al. [10] concluded that almost half of children between 3 and 6 years of age living with HIV-1 had their quality of life affected by oral manifestations of the disease. Massarente et al. [11] observed that children and adolescents with more severe manifestations of AIDS present with a worsened oral health-related quality of life as a result of oral symptoms as well as increased functional, emotional and social limitations.

In view of the limited knowledge regarding this subject, the aim of this study was to investigate the oral health status and its impact on the quality of life of children and adolescents living with HIV-1, who were being followed-up in referral health institutions in the Southern region of the Brazilian Southern state of Santa Catarina.

## Methods

A cross-sectional study involving children and adolescents (n = 36) living with HIV-1 and being followed-up at four public health institutions – centers to HIV treatment referral – in the Southern region of Santa Catarina, Brazil was performed. This study was approved by the ethics committee in human research of the University of the Southern Santa Catarina (protocol number 11.060.4.01.111). As all individuals were younger than 18 years the written informed consent for participation in the study was obtained from a parent or guardian.

A structured questionnaire was used to record information from the medical notes regarding the patient’s sociodemographic profile, such as sex, age, familial income, number of people residing in the family home and level of education. Also, results from laboratory tests, such as CD4+ count and viral load, date of diagnosis, infection route, antiretroviral therapy used were analyzed. Disease progression and presence of HIV-1-associated diseases, such as Tuberculosis, Atypical Mycoplasma, Aspergillosis, Cryptococcal infection, Paracoccidioidomycosis, Pneumonia, Herpes Zoster, Anemia, Cytomegalovirus infection, chronic diarrhea, Toxoplasmosis, Splenomegaly, Lymphopenia, Neuro-toxoplasmosis, Tetanus, thrombocytopenia [12] were also recorded.

Subsequently, an interview with children or their guardian was performed, focusing on the impact of the oral condition on their quality of life, by means of specific questionnaires for each age group. All questionnaires were validated for use in the Portuguese language, in the context of Brazilian culture [13-16] (Table 1). All of them contained questions of different domains, which sought to assess the influence of oral health on the quality of life. The possible answers varied from "never" to "very frequently". The outcome was defined as the presence of impact on quality of life, when at least one frequent or very frequent impact was reported [17].

**Table 1:** Questionnaires used to estimate oral health-related quality of life in children and adolescents

**Questionnaires**	**Age groups**	**Responded**	**Domain**	**Number of questions**	**Validated version in Portuguese**
ECOHIS	1-7 years	Parents/guardians	Child sub-scale; family subscale.	13	Tesch et al. [13]
CPQ_8–10_	8-10 years	Child	Oral symptoms; functional limitations; emotional well-being; social well-being.	29	Barbosa et al. [14]
CPQ_11–14_	11-14 years	Child		16	Torres et al. [15]
OHIP	15-18 years	Adolescent	Functional limitation; physical pain; psychological discomfort; physical incapacity; psychological incapacity; social incapacity; deficiency.	14	Almeida et al. [16]

ECOHIS – Early Childhood Oral Health Impact Scale.

CPQ_8–10_ – Child Perceptions Questionnaire for children aged 8 to 10 years.

CPQ_11–14_– Child Perceptions Questionnaire for children aged 11 to 14 years.

OHIP – Oral Health Impact Profile.

A clinical oral examination was performed to evaluate the presence of dental caries and treatment need, using the DMFT (number of decayed, missing due to caries, or filled permanent teeth) and dmft (number of decayed, missing due to caries, or filled deciduous teeth) indices. The presence of gingival changes, such as linear gingival erythema, pseudomembranous candidosis, erythematous candidosis and angular cheilitis, as well as hairy leukoplakia, intra-oral pigmentation, salivary gland swelling, herpes simplex, recurrent aphthous ulceration, oral verruca, Kaposi’s sarcoma and Non-Hodgkin’s lymphoma were observed according to criteria established in the literature [18-21].

The data obtained were statistically analyzed using SPSS 18.0 software (Chicago, IL, USA). Association studies between outcome and the independent variables were carried out using Fisher’s exact test, with p < 0.05 considered statistically significant.

## Results

Twenty-nine individuals participated in this study and seven individuals refused to participate, giving a response rate of 80.6%. The average age was 10 years (SD = 4.4 years) and the median age was 12 years. Most of the participants were female (58.6%). School attendance was high, with 93.1% going to school. The average monthly income *per capita* was R$ 517.08; SD = R$ 386.09 (approximately US$ 261.15; SD = US$ 194.99) with a median of R$ 333.33 (approximately US$ 168.34).

Regarding HIV-1 infection route, 96.6% came into contact with the virus via vertical transmission. From the total, 82.8% were receiving antiretroviral therapy, of which 25% were using the sugar-rich liquid form. Approximately 82.0% had a CD4+ count higher than 500 cells/mm^3^ and 17.2% had a viral load greater than 10,000 copies/mL. HIV-1-related diseases were present in 16 individuals (55.2%). The most frequent disease being Herpes simplex (50%) followed by Pneumonia (43.7%) (Table 2).

**Table 2:** HIV-1-associated diseases in children and adolescents living with HIV-1 (n = 16)

**Diseases**	n*	**%**
Herpes simplex	8	50.0
Pneumonia	7	43.7
Herpes zoster	2	12.5
Tuberculosis	2	12.5
Anemia	1	6.2
Oral candidosis	1	6.2
Cytomegalovirus infection	1	6.2
Chronic diarrhea	1	6.2
Splenomegaly	1	6.2
Lymphopenia	1	6.2
Neurotoxoplasmosis	1	6.2
Tetanus	1	6.2
Thrombocytopenia	1	6.2

*Some individuals presented with more than one HIV-1-associated disease.

The overall prevalence of dental caries was 75.9% (95% CI 60.3-91.5). In the deciduous dentition it was 63.2% (95% CI 41.5-84.9) and in the permanent dentition it was 58.3% (95% CI 38.6-78.0). The caries indices are described in Table 3. Gingival changes were encountered in 20.7% (95% CI 6.0-35.4) with Linear Gingival Erythema seen in all of these cases. Regarding treatment need, 72.4% (95% CI 56.1-88.7) needed some form of dental treatment, mainly fillings (44.8%).

**Table 3:** Caries indices and components

	**Decayed**	**Missing**	**Filled**	**dmf-t**	**DMF-T**
	$\bar{x}\;\left(\mathrm{SD}\right)$	$\bar{x}\;\left(\mathrm{SD}\right)$	$\bar{x}\;\left(\mathrm{SD}\right)$	$\bar{x}\;\left(\mathrm{SD}\right)$	$\bar{x}\;\left(\mathrm{SD}\right)$
Deciduous dentition	2.63 (2.92)	0.36 (1.38)	0.78 (1.78)	3.70 (4.0)	-
Permanent dentition	2.04 (2.74)	0.40 (0.20)	0.75 (2.13)	-	2.83 (3.43)

$\bar{x}$ = mean.

SD = standard deviation from the mean.

dmf-t = number of decayed, missing due to caries and filled teeth in the primary dentition.

DMF-T = number of decayed, missing due to caries and filled teeth in the permanent dentition.

The prevalence of frequent/very frequent impact on oral health-related quality of life was 69.0% (95% CI 55.2-85.2). The impact varied from 44.4% in pre-school children to 91.7% in those aged between 11 and 14 years (Table 4).

**Table 4:** Prevalence of frequent/very frequent impact on oral health-related quality of life

**Tools**	**Groups**	**Frequent/very frequent impact n (%)**	**Absence of impact n (%)**
ECOHIS	1-7 years	4 (44.4)	5 (55.6)
CPQ_8–10_	8-10 years	3 (75.0)	1 (25.0)
CPQ_11–14_	11-14 years	11 (91.7)	1 (8.3)
OHIP	15-18 years	2 (50.0)	2 (50.0)
**Total**		**20 (69.0)**	**9 (31.0)**

ECOHIS – Early Childhood Oral Health Impact Scale.

CPQ – Child Perceptions Questionnaire.

OHIP – Oral Health Impact Profile.

The results of the associations between frequent/very frequent impact and several independent variables are shown in Table 5. The variables that were associated were age higher than 12 years (p = 0.041) and dental treatment need (p = 0.037).

**Table 5:** Association between frequent/very frequent impact oral health-related quality of life and independent variables (n = 29)

**Variables**	**Frequent/very frequent impact**				**Total**		p* value
	**Yes**		**No**				
	**n**	**%**	**n**	**%**	**n**	**%**	
**Sex**							0.568
Male	8	66.7	4	33.3	12	41.4	
Female	12	70.6	5	29.4	17	58.6	
**Age**							0.041
> 12 years	13	86.7	2	13.3	15	51.7	
≤ 12 years	7	50.0	7	50.0	14	48.3	
**Income *per *** **capita (median)**							0.550
≤ R$ 333.33	10	66.7	5	33.3	15	51.7	
> R$ 333.33	10	71.4	4	28.6	14	48.3	
**Number of people residing in the family home**							0.228
> 4	9	81.8	2	18.2	11	37.9	
≤ 4	11	61.1	7	38.9	18	62.1	
Education level**							0.185
High school	10	83.3	2	16.7	12	50.0	
Elementary school	7	58.3	5	41.7	12	50.0	
**T lymphocyte CD4+**							0.498
≤ 500 (cells/mm^3^)	4	80.0	1	20.0	5	17.2	
> 500 (cells/mm^3^)	16	66.7	8	33.3	24	82.8	
Viral load***							0.448
> 10,000 (copies/mL)	3	60.0	2	40.0	5	17.9	
≤ 10,000 (copies/mL)	17	73.9	6	26.1	23	82.1	
**Time since diagnosis (median)**	9	56.3	7	43.8	16	55.2	0.107
≤ 72 months	11	84.6	2	15.4	13	44.8	
> 72 months							
**Use of antiretroviral**							0.498
Yes	16	66.7	8	33.3	24	82.8	
No	4	80.0	1	20.0	5	17.2	
**Presence of HIV-1-associated disease**							0.647
Yes	11	68.8	5	31.3	16	55.2	
No	9	69.2	4	30.8	13	44.8	
**Presence of caries**							0.108
Yes	17	77.3	5	22.7	22	75.9	
No	3	42.9	4	57.1	7	24.1	
**Dental treatment need**							0.037
Yes	17	81.0	4	19.0	21	72.4	
No	3	37.5	5	62.5	8	27.6	
**Gingival changes**							-
Yes	6	100.0	-	-	6	20.7	
No	14	60.9	9	39.1	23	79.3	

*Fisher’s exact test.

**Two individuals do not go to school and three individuals attend special need schools.

**Data not available for one individual.

## Discussion

It is a fact that the use of antiretroviral therapy has contributed to reduce oral manifestations of HIV-1 in children’s soft tissues. However, chronic use of sugar-rich medications can be considered a relevant factor in the development of dental caries in children living with HIV-1, in addition to the other classic etiological factors of the disease [3,22]. In this study, of the children that used antiretroviral medication, 25.0% still used the liquid form, which contains a large amount of sugar. This fact could be cast as a contributing factor to the high caries prevalence detected. It must also be observed that as children begin treatment immediately after diagnosis, and in the case of vertical transmission within the first months of life, even though the majority are not currently exposed to such liquid medications, they would have had them in the past, which may have contributed to their current caries-related oral health status.

A 2002 study [5] reported a high prevalence of caries and gingival problems in HIV-1 infected children. Ten years later, the present study still detected a high prevalence, despite a decline in prevalence and severity of the disease in Brazilian children [23]. A recent study [24] of 90 individuals living with HIV-1, being followed-up in a pediatric hospital in Mozambique, revealed a mean dmft of 2.6 (SD = 3.6), relatively similar to that found in the present study of 3.1 (SD = 4.0). Regarding the permanent dentition, the values were lower, with a mean of 0.6 (SD = 1.6), compared to 2.83 (SD = 3.43) in this study population. The different age groups studied could have been the reason for the difference reported in the African study. Dental treatment need was high in the African population studied. Restorative treatment was necessary in 44.8% of the individuals. This result was similar to that reported in Nigeria [25] in which 38.2% of 55 children also needed restorative dental treatment.

The onset of gingival and periodontal diseases in individuals infected with HIV-1 is inversely proportional to their immunological status, i.e., as the immune system becomes more compromised, the individual becomes more susceptible to diseases. Portela et al. [26] demonstrated that patients with gingival changes presented leukopenia and severe immunosuppression. The gingival disease present in all cases in this study was the Linear Gingival Erythema.

The prevalence of HIV-1-related oral lesions in this study, detected both similar and contrasting findings in relation to other studies. The most frequent oral lesion was Herpes Simplex, which, unlike Candidosis, has no direct relation with AIDS progression. Its main characteristic is recurrent lesions, which become chronic and form bigger, more widespread and longer lasting lesions [27]. As per the majority of the studies, Candidosis is the most frequent opportunistic infection in individuals living with HIV-1 [3,25]. In fact, approximately 90% of those individuals present with at least one episode of oropharyngeal Candidosis during AIDS development. However, this was not the most prevalent disease in this study sample. This may be due to the fact that in a cross-sectional study, clinical oral examination was performed only once, at which time the individual did not present any *Candida sp* lesions*,* so a future longitudinal study could clarify this question.

Oral health problems have a significant impact on an individuals physical, social and psychological well-being [28]. This becomes even more important in the case of individuals living with HIV-1, especially children and adolescents. In this study, the prevalence of an impact on the quality of life in pre-school children was 44.4%. This result corroborates the findings by Buczinski et al. [10] in a study involving 31 children from three to six years of age, using the ECOHIS as the evaluation tool. The authors concluded that almost half of the children living with HIV-1 had their quality of life affected by oral manifestations of the disease and carious lesions.

In the present study, two variables were found to be statistically associated with oral health-related quality of life. Children and adolescents over 12 years of age reported a higher impact when compared to those under 12. In adolescence, social relationships are intensified, where self-awareness is molded and regulated by the perception that others have, amongst other factors. At this phase of life, physical appearance, including facial, has a high influence in self-satisfaction and social acceptance [29].

The studied individuals who needed dental treatment reported a higher impact when compared to those who did not need treatment. Locker [30] demonstrated that oral disturbances can cause damage and losses that can lead to a self-awareness disorder. It could also lead the individual to experience symptoms of pain and discomfort, functional limitation and dissatisfaction with their appearance. Such situations can produce physical, psychological and social limitations and consequently incapacity. However, dental treatment in individuals living with HIV-1 is no more complex than for other patients. The main objective of dental treatment for patients living with HIV-1 is to improve their quality of life [31]. It is therefore fundamental to scrutinize their medical history and to assess patients expectations regarding their treatment. A lack of initial knowledge of the disease and its clinical aspects, in addition to the prejudice and stigma surrounding AIDS, may lead to serious limitations when treating these patients.

It is important to highlight that the number of participants in this study, though consisting of a sample of children and adolescents living with HIV-1 being followed-up by public services, prevented a more robust analysis to adjust for the role of possible confounding variables on the associations detected. The results of this study should therefore be interpreted with caution. However, other studies involving this type of population also published relatively small cohorts, such as Thoni et al. [32] with 23 children, Buczinski et al. [10] with 31 children and Portela et al. [26] with 35 children.

## Conclusion

Considering the aforementioned limitations, the results of this study allow us to suggest that the children and adolescents living with HIV-1 being followed-up in the public services studied had a high prevalence of caries and high dental treatment need. Almost 76% of children living with HIV-1 in this study presented caries lesions and 72.4% needed dental treatment, while about 56% of HIV-1 negative children at 12 years-old presented at least one caries lesion and 61% demand dental treatment [23]. A high prevalence of frequent/very frequent impact on oral health-related quality of life was also observed. Significant associations between poorer oral health-related quality of life with need for dental treatment and when over 12 years of age demonstrate that despite advances in the care of children living with HIV-1, such as access to drug therapy, the challenges still remain in the areas of prevention and assistance. It is important to highlight the significant social burden associated with this problem, since its sufferers are often stigmatized due to the transmissible and incurable nature of the disease.

Therefore, the orientation of multidisciplinary actions and interventions for health in children and adolescents living with HIV-1 must be focused on the need to improve their quality of life. Incorporating dental care that aims at preventing and controlling oral health issues in the therapeutic planning of such individuals, may contribute to the maintenance of their general health and improve their quality of life. This would generate a more integrated, and therefore more acceptable approach to care from a technical, social and ethical point of view.

## Competing interests

The authors declare that they have no competing interests.

## Authors’ contributions

JT conceived the idea of the study in collaboration with DG, FS-T and JDS. NSR and LSL collected the data and drafted the manuscript. JT performed the statistical analysis and supervised writing. JT and DAN critically reviewed the article. All authors were involved in determining the main contents of the article. All authors read and approved the final manuscript.
